# Combining Theory and Research to Validate a Social Norms Framework Addressing Female Genital Mutilation

**DOI:** 10.3389/fpubh.2021.747823

**Published:** 2022-01-05

**Authors:** Suruchi Sood, Astha Ramaiya

**Affiliations:** ^1^Department of Community Health and Prevention, Dornsife School of Public Health, Drexel University, Philadelphia, PA, United States; ^2^Department of Population, Family and Reproductive Health, Bloomberg School of Public Health, Johns Hopkins University, Baltimore, MD, United States

**Keywords:** FGM, Ethiopia, FGC, interpersonal communication, social norms, attitudes

## Abstract

Female Genital Mutilation (FGM) is a harmful practice with no benefits and considerable harm to girls and women who undergo it. In 2016, the United Nations Joint Program to Eliminate FGM, funded the development and subsequent validation of a monitoring and evaluation framework to understand the relationship between social norms and practicing FGM. Evidence on the framework was gathered through a pilot study in Ethiopia. This paper uses cross-sectional quantitative data from the pilot to operationalize the framework and determine what factors are associated with practicing FGM. A total of 554 and 481 participants answered the question “Have you undergone FGM?” and “Do you know a family member who has undergone FGM?” respectively. Overall, 65% of participants said they had undergone FGM and 32% said they knew someone in their family who had undergone FGM. Predictors of not undergoing FGM included most progressive attitudes vs. less progressive attitudes about FGM and relationship to identity [OR: 1.9 (95% CI: 1.1–3.3)]; region [Afar vs. Addis Ababa: OR: 0.09 (95% CI: 0.02–0.5); Southern Nations Nationalities and People's Regions vs. Addis Ababa: OR: 0.1 (95% CI: 0.05–0.3)], being 36 years old and above vs. 10–19 years (OR: 0.2 (95% CI: 0.1 to 0.7)) and being single, never married vs. married or engaged (OR: 2.8 (95% CI: 1.1–7.0)]. Predictors of knowing a family member who has not undergone FGM included: Higher knowledge vs. lower knowledge [OR: 0.3 (95% CI: 0.1–0.5)]; if the family expected you to abandon FGM, you had a greater odds of knowing a family member who had not undergone FGM [43.6 (95% CI: 2.7–687.8)]; coming from Southern Nations, Nationalities and People's Region was associated with a lower odds of knowing a family member who had not undergone FGM [0.3 (95% CI: 0.1–0.6)]. Being a female influential vs. female caregiver was associated with a higher odds of knowing a family member who had not undergone FGM [2.9 (95% CI: 1.01–5.2)]. This paper has allowed us to validate a theory and research based social norms framework, specifically examining how social and behavior change communication can be used as a mechanism for shifting norms around a given harmful practice. Now that this model has been developed and validated, it is likely to provide a foundation to study the direct and indirect impacts of social norms programming on changing harmful practices, such as FGM.

## Introduction

Female Genital Mutilation (FGM) is a harmful practice with no benefits and considerable harm to girls and women who undergo it. It is prevalent in 30 countries around the globe. There is increasing evidence that the practice continues among immigrant communities in the global north (1). Previous literature has proven that the practice and prevalence of FGM are upheld due to social norms supporting this practice (2–4). An evidence brief prepared by the World Health Organization [WHO in 2019 reported that FGM continued to persist within families and communities due to cultural, religious, and social determinants (5)]. For several decades, interventions have sought to address the myriad social norms surrounding the practice, generating guidance on the process and scale-up of norm-shifting interventions promoting FGM abandonment (6). The lack of rigor and standardization in monitoring and evaluation has made it difficult to determine social and behavior change (SBC) that can be attributed to these norms shifting interventions (7). Recognizing the need for a standard and also adaptable framework to monitor and evaluate SBC communication, the United Nations Joint Program to Eliminate FGM, funded the development and subsequent validation of a monitoring and evaluation framework to fulfill this gap. The resulting framework summarized under the acronym ACT was finalized in 2021 (8). ACT is grounded in multiple individual and social change theories and based on evidence. Evidence on the framework was gathered through a pilot study in Ethiopia. This paper uses the data from the pilot to operationalize the ACT framework. We start with a review of the expansive literature undertaken to develop the framework. Next, we present information on the sampling and methods utilized for a pilot study in Ethiopia. The third section reports the results, which are followed by a discussion of the limitations of this work and recommendations for the future.

## Literature Review

This section summarizes the literature on FGM, social norms theories and measurement, FGM-related social norms, social and behavior change communication (SBCC) as a strategy to shift FGM-related social norms.

### FGM Background

FGM is a traditional practice involving the partial or complete removal of the external female genitalia which has existed for over 5,000 years. FGM is prevalent throughout much of Africa, in parts of the Middle East and Asia, and is an emerging public health issue among immigrant communities in the United States and Europe (9). The World Health Organization defines FGM as “a harmful traditional practice that involves the partial or total removal of external female genitalia or other injuries to female genital organs for non-medical reasons” (10). There are four types of FGM: 1) Clitoridectomy: where the clitoris is partially or totally removed (sometimes this is referred to as sunna); 2) Excision: where the clitoris and labia are partially or totally removed; 3) Infibulation: where the vaginal opening is narrowed by sewing the labia together; and 4) Other: all other forms of FGM procedures, including “pricking, piercing, incising, scraping, and cauterizing the genital area” (11).

FGM affects over 200 million girls and women worldwide and every year 4 million girls are at risk of being subjected to FGM (12). The practice confers no health benefits to those who undergo it, but it is associated with numerous negative physical and mental health outcomes. These negative outcomes can be both short and long-term and include issues such as hemorrhaging, sepsis, shock, HIV, chronic pain, pain with intercourse, prolonged labor, obstetric fistula, and post-traumatic stress disorder (13, 14). The social consequences of undergoing/not undergoing FGM are far-reaching and based on how prevalent the practice is in the community (15). At the society and community level, in areas where FGM is prevalent, girls conforming to social norms are considered faithful, pure, and marriageable but have negative short-term strains on their relationship with their mother. However, after marriage women/girls report traumatic experiences of first sex and distancing from their partner to avoid sex (15). At the individual level, women who have undergone FGM in high prevalence settings experience mental health conditions including depression, anxiety, and post-traumatic stress disorder at a greater rate than women who have not undergone FGM (15). As such, FGM violates the human rights of girls and women.

In 2008, the UNFPA-UNICEF Joint Programme on Eliminating FGM (UNJP) was formed to accelerate the abandonment of FGM, with the goal of eliminating the practice within one generation (16). This is the largest intervention targeting FGM to date and since its inception, over 24 million individuals across 9,000 different communities have made public declarations to abandon FGM, and 3.3 million girls and women have benefitted from FGM prevention and care services across the 17 UNJP countries (17). The UNJP relies on human-rights-based and culturally sensitive approaches to implement SBCC initiatives aimed at changing the social norms upholding FGM.

### Social Norms and FGM

Social scientists have reviewed literature from various disciplines to explicate what social norms are, how they shape behavior, and how they influence individuals and groups (18, 19). While sociologists tend to emphasize the role of norms in defining society and in dictating social behaviors, social psychologists have focused more on why individuals follow social norms (20).

There is overlap in different types of norms. Social norms around FGM as a harmful practice, exist at the intersection of behaviors, beliefs, and expectations. Moral norms (inner conviction of right and wrong) are motivated by conscience rather than by social expectations. Those who have more strongly internalized messaging on the potential health risks linked to FGM are more likely to support ending the practice (21). Religious norms “are distinctive because of their reference to divine command,” [(22), p. 35]. Some supporters abide by FGM as being–sunna, broadly defined as traditional customs and practices within the Islamic community or even required (as a farata) by Islam. In other contexts, FGM is thought to be supported by Christian beliefs. Theorists exploring the normative characteristics of harmful practices such as FGM sometimes equate gender norms and social norms because gender and power codify FGM-related social norms. Gender norms refer to informal rules and shared social expectations that distinguish expected behavior on the basis of gender (20), these cut across all domains of the social-ecological model. For example, they manifest themselves as negative gender role attitudes toward girls and women at the individual level, restrictions on mobility and educational opportunities at the family and community levels, and restrictions such as the age of marriage, emphasis on virginity, and sexual control at the societal level. Attempts to address FGM must account for the individual, social, and structural silencing of women's voices. As such, any measurement of social norm change associated with FGM has to specifically consider gender normative determinants (23). Cislaghi and Heise (24), compare and contrast definitions of these terms and conclude that addressing health equity requires a focus on many social norms that are gender-related (24). Therefore, practitioners have to use both theories from social psychology as well as, the work of feminist scholars.

Two sets of theorizing around social norms are specifically relevant for theory-driven and evidence-based SBC programming. The first set of theories study norms in terms of compliance associated with group dynamics. Norms are considered to be rules or expectations within social groups that guide behaviors and group members expect and are expected to adhere to perceived norms because of social rewards or punishments associated with deviating from the social norms (22, 25). Social convention theory has therefore been used to understand FGM within a social norms perspective (26, 27). This thinking proposes that when sufficient people perform FGM, the practice becomes habitual. Shifting the convention (and sustaining it) requires a critical mass of people allowing their children to marry uncut women and hence abandoning the practice. Although government institutions enforce laws. UNFPA estimates that of the 29 countries in Africa where FGM is traditionally practiced, 26 have laws prohibiting FGM. Fines for practicing FGM in these countries range from 3 months to life imprisonment (28). However, legislative action criminalizing FGM alone does not appear to be a sufficient enough deterrent due to limited knowledge and poor enforcement of legal actions. Based on Rosling's Factfulness framework the one size fits all and zero tolerance of FGM approach actually increases risk and fear (29). Essén and Mosselmans (29) argue that when using the Factfulness framework, FGM is “a dynamic practice, with changes in the practice that is ongoing, and those changes are different in different contexts.” The Factfulness framework provides tools to calculate risk and could aid in limiting stigma that is associated with FGM and allocate resources to health problems based on risk (29).

The second approach situates “norms” as a mediator related to individual and social change within a larger social-ecological (individual, interpersonal, community, institutional, societal, etc.) perspective. This conceptualization of norms has been central in communication studies, with several key theories including social norms as part of a larger SBC equation. This perspective relies on the amalgamation of theories at different levels. At the individual level, the theory of planned behavior developed by Ajzen and Fishbein (30) provides a way to predict intentions and subsequently behavior from an individual's attitudes, perceived behavioral control, and perceived subjective norms (30). On the other hand ideation, social networks and social support theorizing encourage a focus on broader policy decisions and intersectional issues such as gender and religion (31–33).

### ACT Framework

While monitoring and evaluation (M&E) have been a focus of the UNJP since 2008, there is no commonly used and validated methodology for measuring social norms change that could be scaled up. The most robust measurements have relied on data from the Demographic and Health Surveys and the Multiple Indicator Cluster Surveys, which, while examining trends over time at the country level, do not report on the relationship between program implementation and subsequent individual and social change. The UNJP recognized that a rigorous theory and evidence-based M&E framework was needed to link programmatic approaches to the changes observed in order to determine what works, what refinements are needed, what challenges persist, and the overall impact of the work. ACT, a macro-level M&E framework designed to be adaptable while still providing standardization around social norms measurement, was developed to fill this need (32).

**Figure 1:**
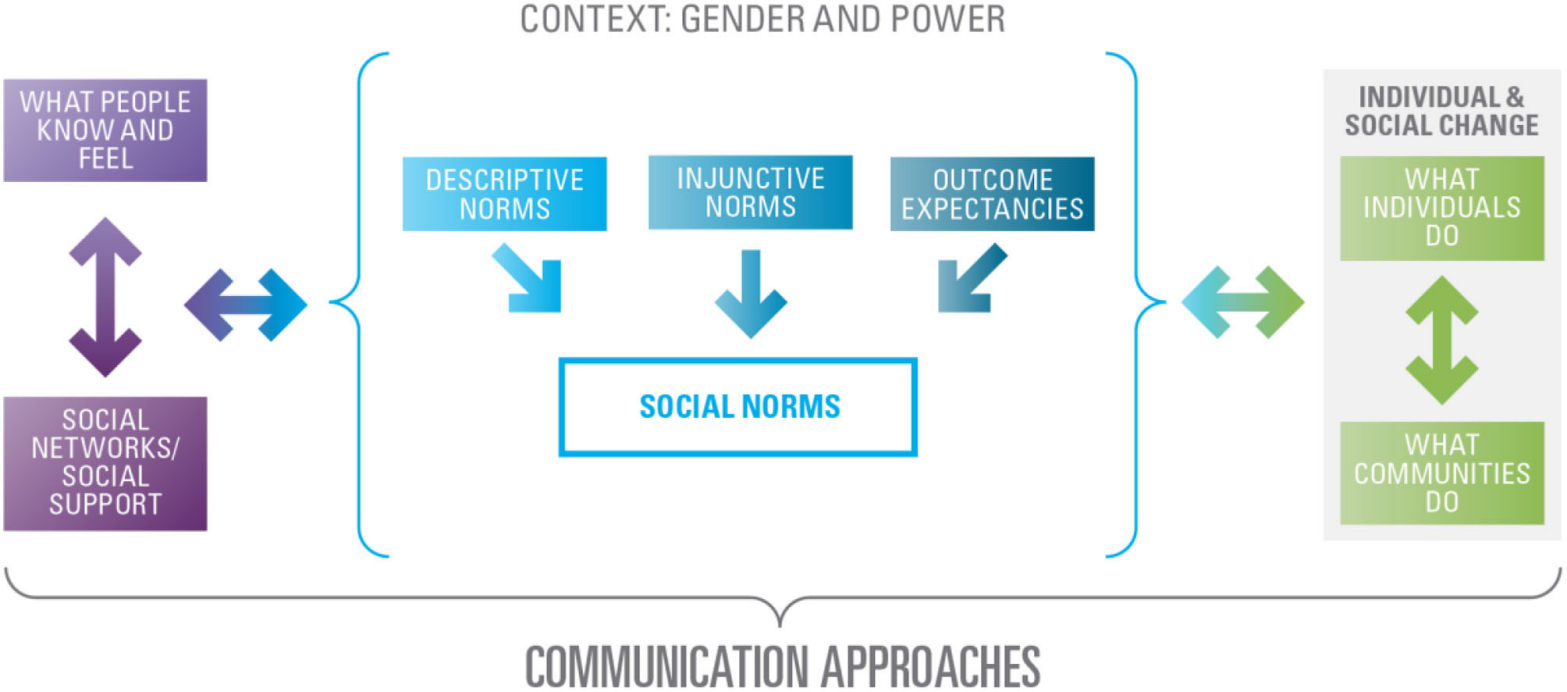
Conceptual model behind the act framework.

The ACT conceptual model is based on the theoretical premise that norms influence thoughts and behaviors and thoughts and behaviors influence norms; if social norms change, then thought and behavior change may ensue and vice versa. Social norms are therefore the intermediary step between what people know and feel their social networks and their social support on the one side and SBC on the other. Two-way arrows indicate the dynamic, multi-directional relationship between norms and other elements of the model. This model incorporates a social-ecological perspective by situating the individual-level factors of knowledge, attitudes, and practices within the broader environmental context, as well as accounting for multiple levels of influence. For example, the model acknowledges that what individuals know and feel are affected by, and in turn affect, their social networks and the level of social support they receive and give. Likewise, individual and social change is a result of the dynamic relationship between what individuals do and what communities do. These constructs all fall under the broader umbrella described as Context: Gender and Power to illustrate the influence that contextual factors have on FGM practices. The entire model is contained within a bracket titled Communication Approaches to signify the influence that SBCC can have on all aspects within the model. Linking the communication approaches within a larger country-level FGM program through ACT allows for measurement of the contribution of and attribution to SBCC (Figure 1).

Table 1 highlights the constructs in the ACT framework. Both the “A” and “C” contain constructs critical to examining social norms change holistically, while the “T” constructs emphasize the larger M&E process within which ACT is housed. The set of indicators for the “A” and “C” constructs are operationalized both quantitatively and qualitatively using questions in the structured interview questionnaire and activities in the focus group discussion (FGD) and in-depth interview (IDI) guides. The Track Individual and Social Change section is critical to linking programmatic efforts to observed changes in the “A” and “C” constructs and have sample indicators associated with quantitative questions in the structured interview tool. These indicators must be customized to fit specific objectives and expected results of specific SBCC efforts. Additionally, this section allows for the examination of the extent to which external validity is hampered by extraneous factors. The second “T” section, triangulate all Data and Analysis, does not contain indicators but instead emphasizes the importance of data triangulation.

**Table 1:** Constructs of the ACT framework.

**Construct: A**	**Construct: C**	**Construct: T**
• Assess What People Know, Feel, and Do • Ascertain Normative Factors	• Consider the Context, Especially Gender & Power • Collect Information on Social Networks and Support	• Track Individual and Social Change • Triangulate all Data and Analysis

## Methods

The conceptual model and ACT framework have gone through many stages of development, review, and validation prior to finalization in 2021. Framework development started with social norms and FGM desk review, which was followed by several meetings with experts in the social norms, communications, and FGM-related fields (34). Instruments were then created to measure the indicators representing the ACT constructs. Following tool development, the UNJP selected Ethiopia as a validation site based on specific criteria (8). The criteria included information on the current prevalence and incidence of FGM, a supportive policy environment, adequate UNJP resources to fund the pilot, and finally research expertise within the country. Guinea was selected as the second pilot study site. Results from the country-level validation in Ethiopia and Guinea were combined to make changes to the global framework (8). Final revisions were based on the suggestions received from the global expert review. The final global framework is accompanied by conceptual definitions of key constructs that comprise social norms, the operationalization of the key constructs, and means of verification through qualitative, quantitative, and participatory tools to measure social norms change (8).

### Study Design and Setting

This manuscript utilizes the cross-sectional quantitative data from the Ethiopia validation study to measure the extent to which the ACT framework addresses social norms related to FGM. The study was conducted in two regions, namely Afar and Southern Nations, Nationalities and Peoples (SNNP), as well as Addis Ababa City Administration. These regions (Afar and SNNP) were purposively selected because the UN Joint Programme (UNJP) was implemented in Afar and SNNP since 2008 and 2014, respectively. Addis Ababa City Administration was also purposively selected for validation in an urban setting. The Addis Ababa City Administration is divided into 10 sub-cities, and of these, Arada sub-city was systematically selected from a random start, and subsequently, woreda 5 was randomly selected out of the 10 woredas in the city. The selection of zones for Afar and SNNP regions was done in consultation with UNJP Ethiopia team. Zone 1 and Zone 3 from Afar region and Kembata-Tembaro zone from SNNP region were purposively selected to collect data from UNJP implementing sites. Taking UNJP implementation phases into consideration, two woredas from Afar region and one woreda from SNNP region using systematic random sampling were selected.

### Sampling

The sample size for any pilot study should broadly reflect the views of the target population but does not have to be representative of the study population as a whole. The sample size must be adequate to provide data that can yield useful insights, but small enough to meet cost, time, and technical constraints. While there are several views on the best sample size for the quantitative pilot, the recommendations generally range between a minimum of 10 individual research participants per group and a maximum of 40 participants (35, 36). Overall, there is some consensus that including 30 individual research participants provides the most robust quantitative estimates (35, 36).

In order to produce statistically reliable estimates of proposed indicators within the proposed study areas. Census maps from the 2007 Population and Housing Census available at the Central Statistics Agency (CSA), were used to identify all enumeration areas (EAs). Sampling was conducted in two stages. At the first stage, the lists of all EAs in sampled woredas were ordered using stratification according to the following variables: zone, woredas, and kebeles, which were further stratified into urban and rural areas. A random stratified sample of EAs was selected as primary sampling units. By taking time and resource constraints into consideration, 10 households were sampled per EA. Dividing the total proposed 120 households by 10, the total number of samples EAs was fixed at 12 (6 EAs per each place of residence, i.e., urban and rural areas). Therefore, a random stratified sample of 4 EAs was selected from each region (Table 2). At the second stage, a list of all households in each selected EAs was prepared using data contained in the 2007 Population and Housing Census EA maps. Trained Frontieri field staff then selected eligible households within each EA. Given that the research team proposed interviewing adolescent girls, their primary caregivers as well as selected influential and social network contacts, the total sample for the structured interviews of the pilot study ranged between 960 and 1,320 (Table 2).

**Table 2:** Planned and actual number of research activities (quantitative and qualitative) conducted, by study areas.

**Study areas and sample**	**Research activities**					
	**Structured interview (SI)**		**IDIs**		**FGDs**	
	**Planned**	**Conducted**	**Planned**	**Conducted**	**Planned**	**Conducted**
Addis Ababa (Total)	440	356	12	12	8	8
Adolescent girl	40	40				
Care giver	40	45				
Social network	240	206				
Community influential	120	65				
SNNP (Total)	440	379	12	12	8	8
Adolescent girl	40	40				
Care giver	40	40				
Social network	240	204				
Community influential	120	95				
Afar (Gewane Woreda) (Total)	220	215	6	6	4	4
Adolescent girl	20	20				
Care giver	20	20				
Social network	120	120				
Community influential	60	55				
Afar (Chifra Woreda) (Total)	220	208	6	6	4	4
Adolescent girl	20	20				
Care giver	20	20				
Social network	120	117				
Community influential	60	51				
Total	1,320	1,158	36	36	24	24

For this research, the primary sample was drawn from eligible households. Household eligibility criteria included that there was an adolescent girl 10–19 years old and a primary caregiver in the household and both provided consent to participating in the study. Both assent and consent procedures were followed for interviewing adolescent girls below 18 years of age. Apart from interviewing both the adolescent girls and their primary caregivers, these respondents were asked to nominate individuals who they considered as influential in matters associated with FGM, as well as, specific individuals in their social networks whose opinion around FGM mattered. Community influential and social network contacts were ranked based on the frequency they were mentioned in each enumeration area. These individuals were interviewed by the data collection team if they were available and consented to be interviewed.

### Variables

This study analyzed a conceptual framework described earlier (Figure 1) which has already been published (32). First, we examined the relationship between knowledge and attitudes on interpersonal communication and Social Support. Knowledge was operationalized based on four questions: (1) “In some countries there are traditional practices that may be harmful to girls and women. Have you heard of any such traditional practices? What are they?” (2) “In some countries, there is a practice in which a girl may have part of her genitals cut. Have you ever heard about this practice?” (3) “Can you describe the different ways a girl can be cut/ excised?” And (4) “Using the following images, can you identify the type of cutting?” A composite was created from 0 to 8 for these four questions. However, because the data were skewed, participants who had 0–3 were categorized as “lower knowledge” and participants who had 4–8 were categorized as “higher knowledge.”

Attitudes were operationalized as attitudes about power and gender, attitudes about FGM and relationship to identity, and attitudes about FGM and religion. Attitudes about power and gender were made into a composite and then categorized based on the distribution of the data. The questions which measured attitudes about power and gender included: “FGM teaches girls to be obedient”; “FGM ensures that girls retain their cleanliness”; “FGM ensures that girls remain pure before marriage”; “FGM ensures that girls retain their femininity”; “Girls can be socialized even without undergoing FGM” and “FGM teaches girls to be respectful.” The codes were reversed for the first question and the last question. Girls with the most progressive attitudes were coded as 1 and those with lesser attitudes were coded as 0. Attitudes about FGM and relationship to identity was a composite of three questions “FGM has always been a part of our traditions,” “FGM is a part of our culture” and “FGM is part of our identity.” The codes for all three were reversed and those with the highest score were coded as 1 and those with less than that were coded as 0. Lastly, attitudes about FGM and religion were based on one question “It is a religious duty to perform FGM,” girls who disagreed were coded as 1, and girls who agreed/neutral were coded as 0.

IPC was operationalized by the question: “Who have you engaged in a conversation about topics related to FGM with?” this was multiple select options and a composite was created with the number of people they talked to FGM about. Since the distribution of the data was skewed toward 0, we created a dichotomous variable with 0 being operationalized as 0 and 1 to 9 being operationalized as 1. Social support was dichotomized based on two questions: “Who do you turn to for advice about FGM?” and “Who do you turn to for help (beyond advice i.e., supplies, money, transportation) about FGM?” Similar to IPC, this was a multiple select question and participants selected the people they turned to for advice and help. We dichotomized this variable with 0 representing neither seeking advice and help and 1 representing seeking advice and/or help from others.

Finally, we looked at the relationship between knowledge, attitudes, IPC, SS, social norms, and behavior. Behavior was operationalized based on two questions: “Have you undergone FGM?” and “Do you know a family member who has undergone FGM?” Both these questions were dichotomous with an answer choice of yes or no. Participants who said yes were coded as 0, and participants who said no were coded as 1.

Confounders in our regression models included region (Addis Ababa, Afar, and Sothern Nations Nationalities and People's Region), respondent type (adolescent girls, female caregiver, female influential and female social network), age (categorized as 10–19, 20–35, and 36 and above), marital status (married or engaged; widowed, divorced or separated and single, never married) religion (categorized as Christian and Muslim), educational status (no formal education, primary education, and secondary education and higher) and socioeconomic status. Socio-economic status was made into a composite based on the main source of drinking water, toilet facility, and floor material of the house. Thereafter, a tertile was created which was categorized as “most poor,” “poor,” and “least poor.”

### Analysis

**Figure 2:**
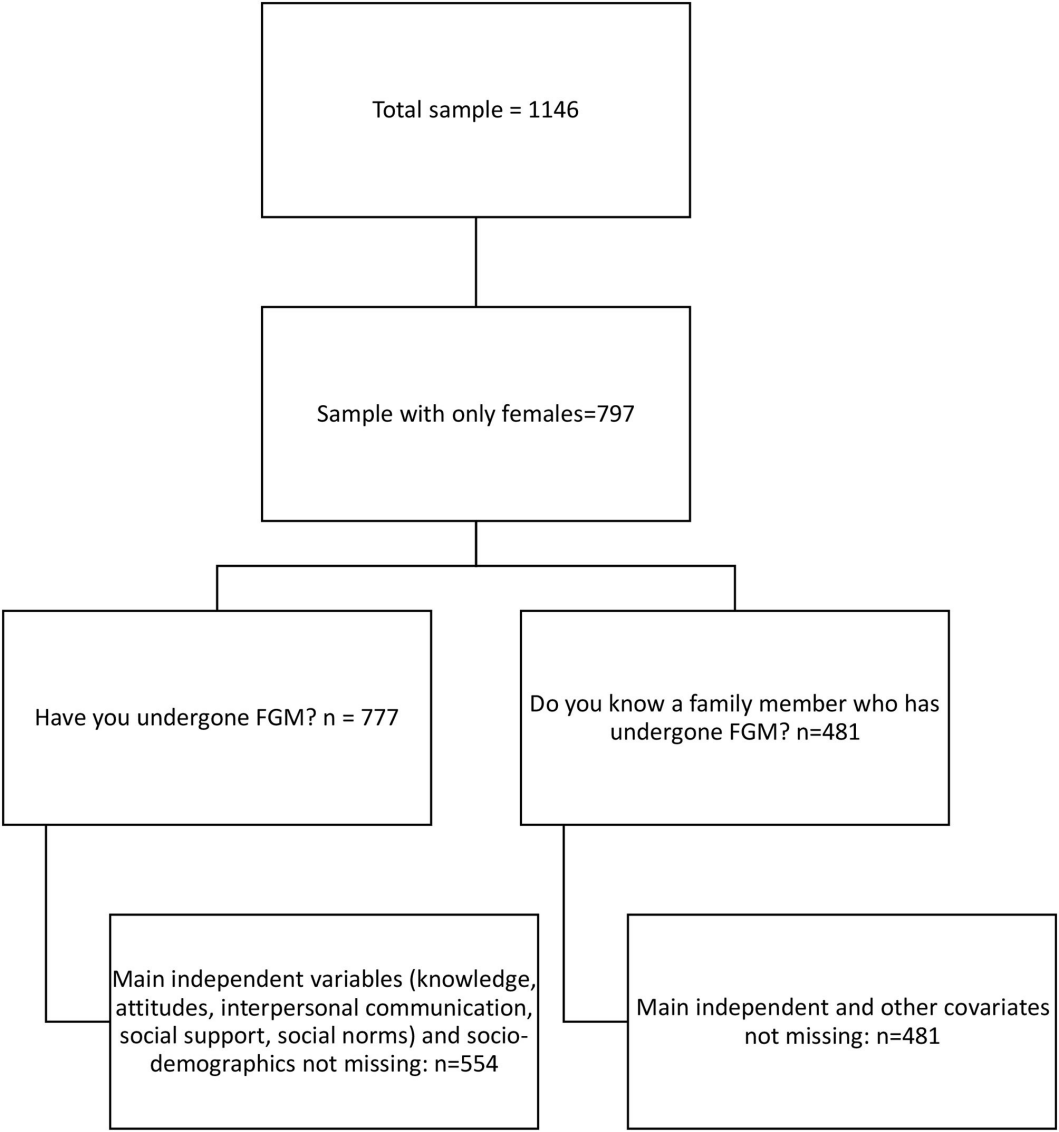
Flow chart of final analytical sample.

Although the overall questionnaire collected information from men, women, girls, and boys; only girls and women were included for this analysis because our main dependent variable asked if they had undergone FGM. Figure 2 shows how the final analytical sample was determined.

STATA 14.2 was used for all descriptive, bivariate and multivariable analyses. A univariate table was created to describe the population. Thereafter, seven multivariable analyses were conducted to determine the relationships portrayed in the conceptual model:

1. In the first two multivariable regressions, we looked at the relationship between knowledge and attitudes on interpersonal communication and social support.
2. In the second to fifth multivariable regressions, we looked at the relationship between knowledge, attitudes, interpersonal communication, and social support on social norms (descriptive norms, injunctive norms, rewards, and punishments).
3. In the sixth and seventh multivariable regressions, we looked at the relationship between knowledge, attitudes, IPC, SS, social norms, and behavior (have they undergone FGM, and do they know someone in their family who has undergone FGM?).

For all multivariable regressions, we used logistic regressions to determine the association between the variables after controlling for all other variables. Adjusted odds ratios with 95% confidence intervals are reported below.

## Results

Table 3 compares the socio-demographic characteristics between participants who were in the final sample and those who were dropped. Participants who were excluded were more likely to be young, never married social network contacts in the SNNP region.

**Table 3:** Comparison of socio-demographics between participants included and not included.

**Socio-demographic characteristics**	**Overall (*n* = 777)**	**Included participants (*n* = 554)**	**Not included participants (*n* = 223)**
**Region**			
Addis Ababa	34.4%	37.4%	26.9%
Afar	36.7%	37.4%	35.0%
Southern Nations, Nationalities and People's Regions	29.0%	25.3%	38.1%^***^
**Respondent**			
Adolescent girl	15.4%	11.7%	24.7%
Female caregiver	14.2%	15.3%	11.2%
Female influential	13.0%	14.3%	9.9%
Female social network	57.4%	58.7%	54.3%^***^
**Age**			
10–19 years	34.8%	27.8%	52.3%
20–35 years	37.0%	40.8%	27.3%
36 and over	28.3%	31.4%	20.5%^***^
**Marital status**			
Married or engaged	48.5%	54.5%	33.6%
Widowed, divorced or separated	10.9%	12.3%	7.6%
Single, never married	40.5%	33.2%	58.7%^***^
**Religion**			
Christian (Orthodox, Protestant, Catholic)	60.6%	59.2%	64.1%
Muslim	39.4%	40.8%	35.9%
**Educational status**			
No formal education	34.8%	37.4%	28.3%
Primary education	38.2%	33.4%	50.2%
Secondary education and higher	27.0%	29.2%	21.5%^***^
**Socio-economic status**			
Tertile 1 (most poor)	43.7%	42.4%	46.9%
Tertile 2 (poor)	25.5%	24.2%	28.8%
Tertile 3 (least poor)	30.8%	33.4%	24.3%^*^

* *p ≤ 0.05*,

*** *p ≤ 0.001*.

Table 4 looks at the socio-demographic and key variable characteristics of the sample. Overall, in terms of the socio-demographic, there was an equal distribution of the participants across the regions (37% in Addis Ababa and Afar; 25% in Southern Nations, Nationalities and People's Regions). 72% of the participants were 20 years and older. A little more than half were married or engaged (55%); 60% were Christian; 37% had no formal education and 42% were in the lowest tertile of socio-economic status. There were significant differences between respondents by age, marital status, and education status. In comparison to adolescent girls: female caregivers, female influential and female social network were all adults (20 years and older); married, and had no formal education.

**Table 4:** Socio-demographics and key variable characteristics by respondent.

**Socio-demographic and key variable characteristics**	**Overall (*n* = 554)**	**Adolescent girl** **(*n* = 65)**	**Female caregiver** **(*n* = 85)**	**Female influential** **(*n* = 79)**	**Female social network** **(*n* = 325)**
**Region**					
Addis Ababa	37.4%	38.5%	37.7%	39.2%	36.6%
Afar	37.4%	35.4%	37.7%	27.9%	40.0%
Southern Nations, Nationalities and People's Regions	25.3%	26.2%	24.7%	32.9%	23.4%
**Age**					
10–19 years	27.8%	100%	0%	5.1%	26.2%
20–35 years	40.8%	0%	49.4%	45.6%	45.5%
36 and over	31.4%	0%	50.6%	49.4%	28.3%^***^
**Marital status**					
Married or engaged	54.5%	6.2%	77.7%	63.3%	56.0%
Widowed, divorced or separated	12.3%	0%	20.0%	19.0%	11.1%
Single, never married	33.2%	93.9%	2.4%	17.7%	32.9%^***^
**Religion**					
Christian (Orthodox, Protestant, Catholic)	59.2%	56.9%	58.8%	70.9%	56.9%
Muslim	40.8%	43.1%	41.2%	29.1%	43.1%
**Educational status**					
No formal education	37.4%	6.2%	63.5%	39.2%	36.3%
Primary education	33.4%	61.5%	20.0%	26.6%	32.9%
Secondary education and higher	29.2%	32.3%	16.5%	34.2%	30.8%^***^
**Socio-economic status**					
Tertile 1 (most poor)	42.4%	43.1%	47.1%	32.9%	43.4%
Tertile 2 (poor)	24.2%	23.1%	20.0%	32.9%	23.4%
Tertile 3 (least poor)	33.4%	33.9%	32.9%	34.2%	33.2%
**Knowledge**					
Lower	67.7%	86.2%	52.9%	64.6%	68.6%
Higher	32.3%	13.9%	47.1%	35.4%	31.4%^***^
**Attitudes about power and gender**					
Not as progressive	55.6%	40.0%	65.9%	48.1%	57.9%
Most progressive	44.4%	60.0%	34.1%	51.9%	42.2%^**^
**Attitudes about FGM and relationship to identity**					
Not as progressive	66.4%	52.3%	65.9%	76.0%	67.1%
Most progressive	33.6%	47.7%	34.1%	24.1%	32.9%^*^
**Attitude about FGM and religion**					
Agree or have neutral attitude on “it is a religious duty to perform FGM”	29.1%	16.9%	31.8%	29.1%	30.8%
Disagree that there is a religious duty to perform FGM	70.9%	83.1%	68.2%	70.9%	69.2%
**Engaged in a conversation about FGM**					
No	52.7%	60.0%	55.3%	50.6%	51.1%
Yes	47.3%	40.0%	44.7%	49.4%	48.9%
**Social support (sought advice or instrumental support surrounding FGM)**					
No	9.0%	6.2%	9.4%	8.9%	9.5%
Yes	91.0%	93.9%	90.6%	91.1%	90.5%
**Descriptive norms**					
**Family decision to abandon FGM**					
No	31.2%	24.6%	34.1%	26.6%	32.9%
Yes	68.8%	75.4%	65.9%	73.4%	67.1%
**Other people in the community decide to abandon FGM**					
No	32.0%	24.6%	34.1%	25.3%	34.5%
Yes	68.1%	75.4%	65.9%	74.7%	65.5%
**Society in general decides to abandon FGM**					
No	32.5%	32.3%	34.1%	25.3%	33.9%
Yes	67.5%	67.7%	65.9%	74.7%	66.2%
**Injunctive norms**					
**Family expects you to abandon FGM**					
No	32.1%	26.2%	34.1%	26.6%	34.2%
Yes	67.9%	73.9%	65.9%	73.4%	65.9%
**Other people in the community expect you to abandon FGM**					
No	31.4%	23.1%	34.1%	25.3%	33.9%
Yes	68.6%	76.9%	65.9%	74.7%	66.2%
**Society in general expect you to abandon FGM**					
No	32.5%	32.3%	36.5%	25.3%	33.2%
Yes	67.5%	67.7%	63.5%	74.7%	66.8%
**Punishments identified with abandoning FGM**					
Yes	36.5%	27.7%	38.8%	29.1%	39.4%
No	63.5%	72.3%	61.2%	70.9%	60.6%
**Rewards identified with abandoning FGM**					
No	42.1%	32.3%	38.8%	46.8%	43.7%
Yes	57.9%	67.7%	61.2%	53.2%	56.3%
**Personally undergone FGM**					
Yes	65.2%	44.6%	82.4%	63.3%	65.2%
No	34.8%	55.4%	17.7%	36.7%	34.8%^***^
**Know someone in their family who have undergone FGM (Not asked to adolescent girls)**	***N*** **=** **481**		***N*** **=** **85**	***N*** **=** **77**	***N*** **=** **319**
Yes	32.0%		41.2%	26.0%	31.0%
No	68.0%		58.8%	74.0%	69.0%^∧^

∧ *p ≤ 0.1*

* *p ≤ 0.05*,

** *p ≤ 0.01*,

*** *p ≤ 0.001*.

*□ :Adolescent girls not asked this question*.

Next, we assessed knowledge about FGM, attitudes toward FGM, interpersonal communication, social support, and social norms. Overall, 32% had higher knowledge about FGM; 44% had more progressive attitudes about power and gender; 34% had more progressive attitudes about FGM and its relationship to identity; 71% disagreed that there is a religious duty to perform FGM; 47% engaged in a conversation about FGM; 91% sought advice or instrumental support surrounding FGM. In terms of descriptive norms; approximately two-thirds of the respondents said their family, other people in the community, and society, in general, had made a decision to abandon FGM, respectively. A similar proportion of respondents reported following injunctive norms, by stating that their family, other people in the community, and society, in general, expected them to abandon FGM. For rewards and punishments, 64% did not identify any punishments with abandoning FGM; 57% identified rewards with abandoning FGM. There were significant differences by respondent type for knowledge, attitudes about power and gender, attitudes about FGM, and its relationship to identity. Adolescent girls had significantly lower knowledge but more progressive attitudes about FGM and its relationship to identity in comparison to the other respondent groups. Female caregivers and female social networks had fewer progressive attitudes about power and gender in comparison to adolescent girls and female influentials.

Lastly, we assessed behaviors by respondent type. Overall, 65% of participants said they had undergone FGM and 32% said they knew someone in their family who had undergone FGM. Adolescent girls reported a significantly lower proportion of getting cut in comparison to the other respondent groups.

Table 5 assesses the effect of knowledge and attitudes on interpersonal communication and social support. Predictors of interpersonal communication included region and marital status. Participants from Afar and Southern Nations, Nationalities and People's Region's had a 7.1 (95% CI: 2.1–24.5) and 2.2 (95% CI: 1.1–4.5) greater odds of talking about FGM in comparison to participants from Addis Ababa, respectively. Participants who were widowed, divorced or separated had 0.2 times (95% CI: 0.1–0.5) lower odds of talking about FGM in comparison to participants who were married or engaged. Socio-economic status had a relationship with social support. Participants who were in tertile 2 (poor), had 0.3 times lower odds (95% CI: 0.2–0.7) of seeking social support in comparison to participants who were most poor. No other predictors were significantly associated with interpersonal communication and social support.

**Table 5:** Relationship between knowledge and attitudes on interpersonal communication and social support.

	**Interpersonal communication (IPC) [Odds ratio (OR) (95% CI)] (*n* = 554)**	**Social support [OR (95% CI)] (*n* = 554)**
**Knowledge**		
Lower	-ref-	-ref-
Higher	1.1 (0.7–1.9)	0.8 (0.4–1.7)
**Attitudes about power and gender**		
Not as progressive	-ref-	-ref-
Most progressive	0.9 (0.5–1.4)	1.4 (0.6–3.1)
**Attitudes about FGM and relationship to identity**		
Not as progressive	-ref-	-ref-
Most progressive	0.7 (0.4–1.2)	2.0 (0.8–5.0)
**Attitude about FGM and religion**		
Agree or have neutral attitude on “it is a religious duty to perform FGM”	-ref-	-ref-
Disagree that there is a religious duty to perform FGM	1.5 (0.7–3.1)	1.4 (0.4–4.2)
**Region**		
Addis Ababa	-ref-	-ref-
Afar	7.1 (2.1–24.5)^**^	2.5 (0.5–13.3)
Southern Nations, Nationalities and People's Regions	2.2 (1.1–4.5)^*^	0.9 (0.3–2.5)
**Respondent**		
Adolescent girl	-ref-	-ref-
Female caregiver	0.5 (0.2–1.4)	0.8 (0.2–3.9)
Female influential	1.1 (0.4–2.8)	0.9 (0.2–4.4)
Female social network	0.9 (0.4–1.8)	0.7 (0.2–2.5)
**Age**		
10–19 years	-ref-	-ref-
20–35 years	1.3 (0.6–3.2)	2.6 (0.5–14.4)
36 and over	1.2 (0.5–3.3)	1.1 (0.2–6.4)
**Marital status**		
Married or engaged	-ref-	-ref-
Widowed, divorced or separated	0.2 (0.1–0.5)^***^	1.2 (0.4–3.2)
Single, never married	0.6 (0.3–1.3)	1.4 (0.3–7.2)
**Religion**		
Christian (Orthodox, Protestant, Catholic)	-ref-	-ref-
Muslim	1.5 (0.5–4.5)	0.4 (0.1–1.9)
**Educational status**		
No formal education	-ref-	-ref-
Primary education	0.6 (0.3–1.0)^∧^	1.3 (0.5–3.2)
Secondary education and higher	0.6 (0.3–1.2)	1.6 (0.5–4.6)
**Socio-economic status**		
Tertile 1 (most poor)	-ref-	-ref-
Tertile 2 (poor)	0.9 (0.5–1.6)	0.3 (0.2–0.7)^**^
Tertile 3 (least poor)	1.2 (0.5–2.7)	0.9 (0.2–3.0)

∧ *p ≤ 0.1*,

* *p ≤ 0.05*,

** *p ≤ 0.01*,

*** *p ≤ 0.001*.

Table 6 looks at the effect of knowledge, attitudes, social support, and interpersonal communication on social norms. Factors that had a relationship with two or more descriptive norms and two or more injunctive norms included attitudes about power and gender, attitudes about FGM and religion, engaging in a conversation about FGM, region, and education. Participants with the most progressive attitudes vs. less progressive attitudes about power and gender had a 3.4 (95% CI: 1.1–9.9), 3.8 (95% CI: 1.4–10.0), and 8.6 (95% CI: 2.8–26.4) greater odds of stating that their family, other people in the community and society, in general, made a decision to abandon FGM, respectively. Additionally, these participants had a greater odds of reporting that other people in the community expected them to abandon FGM [3.8 (95% CI: 1.3–10.8)] and society in general expected them to abandon FGM [7.7 (95% CI: 2.6–22.7)]. Participants who disagreed that there was a religious duty to perform FGM vs. those who agreed had a 28.9 (95% CI: 9.4–89.3), 16.3 (95% CI: 6.0–43.8), and 32.1 (95% CI: 9.5–108.1) greater odds of stating that their family, other people in the community and society, in general, made a decision to abandon FGM, respectively. This relationship held true for injunctive norms as well, where participants who disagreed vs. agreed that there was a religious duty to perform FGM, had greater odds of saying that the family [26.5 (95% CI: 9.2–76.3)], other people in the community [16.9 (95% CI: 6.0–47.4)] and society in general [19.9 (95% CI: 6.7–58.9)] expected them to abandon FGM. Participants who had engaged in a conversation about FGM vs. not had 3.7 times (95% CI: 1.2–11.9) and 7.8 times (95% CI: 2.2–27.6) greater odds of reporting their family and society, in general, made a decision to abandon FGM, respectively. Additionally, these participants also reported a greater odds of family [3.0 (95% CI: 1.04–8.6)], other people in the community [3.2 (95% CI: 1.1–9.6)] and society in general [4.2 (95% CI: 1.4–12.8)] expecting them to abandon FGM.

**Table 6:** Effect of knowledge, attitudes, interpersonal communication, and social support on social norms.

	**Descriptive norms [OR (95% CI)] (*****n*** **=** **554)**			**Injunctive norms [OR (95% CI)] (*****n*** **=** **554)**			**Punishments [OR (95% CI)] (*n* = 554)**	**Rewards [OR (95% CI)] (*n* = 554)**
	**Family decision to abandon FGM**	**Other people in the community decide to abandon FGM**	**Society in general decides to abandon FGM**	**Family expects you to abandon FGM**	**Other people in the community expect you to abandon FGM**	**Society in general expect you to abandon FGM**	**No punishments identified with abandoning FGM**	**Rewards identified with abandoning FGM**
**Knowledge**								
Lower	-ref-	-ref-	-ref-	-ref-	-ref-	-ref-	-ref-	-ref-
Higher	1.8 (0.6–5.2)	1.0 (0.4–2.5)	0.8 (0.3–2.4)	1.5 (0.6–4.1)	1.2 (0.5–3.1)	1.1 (0.4–2.9)	2.2 (1.3–3.8)^**^	0.6 (0.4–0.9)^*^
**Attitudes about power and gender**								
Not as progressive	-ref-	-ref-	-ref-	-ref-	-ref-	-ref-	-ref-	-ref-
Most progressive	3.4 (1.1–9.9)^*^	3.8 (1.4–10.0)^**^	8.6 (2.8–26.4)^***^	2.4 (0.9–6.3)^∧^	3.8 (1.3–10.8)^*^	7.7 (2.6–22.7)	1.5 (0.9–2.5)	1.6 (1.02–2.6)^*^
**Attitudes about FGM and relationship to identity**								
Not as progressive	-ref-	-ref-	-ref-	-ref-	-ref-	-ref-	-ref-	-ref-
Most progressive	4.2 (1.1–15.8)^*^	3.0 (1.0–9.3)^∧^	1.5 (0.5–5.2)	4.4 (1.4–13.7)^*^	3.2 (1.0–11.0)^∧^	1.4 (0.5–4.6)	1.3 (0.8–2.2)	1.6 (1.0–2.8)^∧^
**Attitude about FGM and religion**								
Agree or have neutral attitude on “it is a religious duty to perform FGM”	-ref-	-ref-	-ref-	-ref-	-ref-	-ref-	-ref-	-ref-
Disagree that there is a religious duty to perform FGM	28.9 (9.4–89.3)^***^	16.3 (6.0–43.8)^***^	32.1 (9.5–108.1)^***^	26.5 (9.2–76.3)^***^	16.9 (6.0–47.4)^***^	19.9 (6.7–58.9)^***^	1.3 (0.6–2.7)	2.2 (1.1–4.3)^*^
**Engaged in a conversation about FGM**								
No	-ref-	-ref-	-ref-	-ref-	-ref-	-ref-	-ref-	-ref-
Yes	3.7 (1.2–11.9)^**^	2.7 (1.0–7.4)	7.8 (2.2–27.6)	3.0 (1.04–8.6)^*^	3.2 (1.1–9.6)^*^	4.2 (1.4–12.8)^*^	0.6 (0.4–0.9)^*^	1.6 (1.0–2.7)^∧^
**Social support (sought advice or instrumental support surrounding FGM)**								
No	-ref-	-ref-	-ref-	-ref-	-ref-	-ref-	-ref-	-ref-
Yes	0.3 (0.1–1.5)	0.4 (0.1–1.5)	0.3 (0.1–1.6)	0.3 (0.1–1.3)	0.4 (0.1–1.7)	0.4 (0.1–1.9)	0.5 (0.2–1.2)	1.8 (0.9–3.8)
**Region**								
Addis Ababa	-ref-	-ref-	-ref-	-ref-	-ref-	-ref-	-ref-	-ref-
Afar	0.1 (0.01–0.8)^*^	0.1 (0.01–0.7)^*^	0.01 (0.001–0.2)^***^	0.1 (0.01–0.9) ^*^	0.1 (0.01–0.6)^*^	0.02 (0.001–0.2)^***^	0.1 (0.02–0.3)^***^	0.6 (0.2–2.1)
Southern Nations, Nationalities and People's Regions	0.2 (0.04–1.4)	0.2 (0.03–0.8)^*^	0.02 (0.001–0.2)^**^	0.7 (0.1–3.1)	0.1 (0.02–0.8)^*^	0.05 (0.007–0.4)^**^	0.5 (0.2–1.1)	4.7 (2.2–10.0)^***^
**Respondent**								
Adolescent girl	-ref-	-ref-	-ref-	-ref-	-ref-	-ref-	-ref-	-ref-
Female caregiver	3.1 (0.4–21.7)	2.7 (0.5–15.3)	15.0 (2.1–106.1)^**^	3.2 (0.5–20.3)	2.2 (0.4–14.2)	10.9 (1.7–70.9)^*^	0.7 (0.3–1.8)	1.2 (0.5–3.1)
Female influential	3.9 (0.5–32.3)	3.5 (0.5–22.9)	13.9 (1.5–127.5)^*^	4.8 (0.7–33.8)	2.8 (0.4–21.4)	15.7 (1.9–128.0)^**^	1.1 (0.4–3.1)	0.7 (0.3–1.7)
Female social network	2.1 (0.5–9.4)	1.4 (0.4–5.6)	5.9 (1.4–24.4)^*^	1.9 (0.5–7.8)	1.3 (0.3–5.5)	8.6 (2.0–36.2)^**^	0.8 (0.3–1.7)	0.8 (0.4–1.8)
**Age**								
10–19 years	-ref-	-ref-	-ref-	-ref-	-ref-	-ref-	-ref-	-ref-
20–35 years	1.3 (0.1–14.1)	1.3 (0.2–9.9)	2.9 (0.3–27.2)	0.6 (0.1–4.2)	1.2 (0.1–10.0)	1.7 (0.2–17.0)	0.9 (0.4–2.2)	1.1 (0.5–2.5)
36 and over	0.7 (0.1–9.1)	1.1 (0.1–9.7)	1.4 (0.1–15.7)	0.4 (0.04–3.2)	1.0 (0.1–10.5)	1.1 (0.1–12.2)	0.7 (0.3–1.9)	1.1 (0.4–2.7)
**Marital status**								
Married or engaged	-ref-	-ref-	-ref-	-ref-	-ref-	-ref-	-ref-	-ref-
Widowed, divorced or separated	1.7 (0.4–6.6)	1.0 (0.3–3.4)	1.3 (0.3–5.7)	1.6 (0.5–5.7)	1.2 (0.3–4.5)	1.7 (0.4–6.6)	0.9 (0.5–1.8)	1.4 (0.7–2.6)
Single, never married	1.0 (0.1–10.7)	0.9 (0.1–6.8)	1.8 (0.2–15.1)	0.5 (0.1–3.3)	1.0 (0.1–8.1)	2.3 (0.3–20.6)	1.2 (0.6–2.8)	1.1 (0.5–2.2)
**Religion**								
Christian (Orthodox, Protestant, Catholic)	-ref-	-ref-	-ref-	-ref-	-ref-	-ref-	-ref-	-ref-
Muslim	0.2 (0.01–1.6)	0.2 (0.02–0.98)^*^	0.1 (0.004–2.4)	0.4 (0.1–2.5)	0.1 (0.02–0.8)	0.3 (0.02–3.2)	1.0 (0.3–3.2)	2.2 (0.7–6.7)
**Educational status**								
No formal education	-ref-	-ref-	-ref-	-ref-	-ref-	-ref-	-ref-	-ref-
Primary education	4.0 (1.1–14.2)^*^	3.4 (1.1–10.5)^*^	5.5 (1.4–21.5)^*^	2.5 (0.8–8.0)	3.8 (1.2–12.6)^*^	3.5 (1.02–12.1)^*^	0.7 (0.4–1.4)	1.1 (0.6–2.1)
Secondary education and higher	3.6 (0.8–16.4)	4.2 (1.04–16.4)^*^	5.4 (1.1–27.7)^*^	3.1 (0.7–12.5)	4.9 (1.1–21.2)^*^	5.9 (1.2–28.5)^*^	0.5 (0.3–1.1)^∧^	1.0 (0.5–1.9)
**Socio-economic status**								
Tertile 1 (most poor)	-ref-	-ref-	-ref-	-ref-	-ref-	-ref-	-ref-	-ref-
Tertile 2 (poor)	0.8 (0.3–2.3)	0.8 (0.3–2.2)	0.9 (0.3–2.5)	1.0 (0.4–2.8)	0.8 (0.3–2.2)	0.8 (0.3–2.2)	0.8 (0.4–1.5)	0.7 (0.4–1.4)
Tertile 3 (least poor)	1.4 (0.2–8.6)	0.5 (0.1–2.2)	0.7 (0.1–4.2)	1.7 (0.3–8.6)	0.4 (0.1–2.2)	0.5 (0.1–2.6)	0.4 (0.2–1.01)^^∧^^	1.2 (0.5–2.7)

∧ *p ≤ 0.1*,

* *p ≤ 0.05*,

** *p ≤ 0.01*,

*** *p ≤ 0.001*.

Predictors of identifying no punishments with abandoning FGM and rewards identified with abandoning FGM included knowledge, attitudes about power and gender, attitude about FGM and religion, and engaging in a conversation about FGM. Higher knowledge vs. lower increased odds of stating there were no punishments for abandoning FGM [2.2 (95% CI: 1.3–3.8)] but lower odds with identifying rewards to abandon FGM [0.6 (95% CI: 0.4–0.9)]. Most progressive attitudes about power and gender vs. not as progressive increased odds of identifying rewards of abandoning FGM [1.6 (95% CI: 1.02–2.6)]. Disagreeing that there was a religious duty to perform FGM vs. agreeing was associated with a greater odds of identifying rewards [2.2 (95% CI: 1.1–4.3)]. Engaging in a conversation about FGM was associated with a lower odds of stating there were no punishments identified with abandoning FGM [0.6 (95% CI: 0.4–0.9)].

Table 7 looks at the relationship between knowledge, attitudes, IPC, social support, and social networks on two behaviors: have not undergone FGM and family member has not undergone FGM. Predictors of not undergoing FGM included most progressive attitudes vs. less progressive attitudes about FGM and relationship to identity [1.9 (95% CI: 1.1–3.3)]; region [Afar vs. Addis Ababa: 0.09 (95% CI: 0.02–0.5); Southern Nations Nationalities and People's Regions vs. Addis Ababa: 0.1 (95% CI: 0.05–0.3)], being 36 years old and above vs. 10–19 years [0.2 (95% CI: 0.1–0.7)] and being single, never married vs. married or engaged [2.8 (95% CI: 1.1–7.0)].

**Table 7:** Effect of knowledge, attitudes, IPC, social support, and social norms on behavior.

	**Have not undergone FGM [OR (95% CI)] (*n* = 554)**	**Family member has not undergone FGM [OR (95% CI)] (*n* = 481)**
**Knowledge**		
Lower	-ref-	-ref-
Higher	1.3 (0.6–2.6)	0.3 (0.1–0.5)^***^
**Attitudes about power and gender**		
Not as progressive	-ref-	-ref-
Most progressive	0.9 (0.5–1.7)	1.2 (0.6–2.1)
**Attitudes about FGM and relationship to identity**		
Not as progressive	-ref-	-ref-
Most progressive	1.9 (1.1–3.3)^*^	1.1 (0.6–2.1)
**Attitude about FGM and religion**		
Agree or have neutral attitude on “it is a religious duty to perform FGM”	-ref-	-ref-
Disagree that there is a religious duty to perform FGM	1.4 (0.4–5.2)	1.3 (0.5–3.4)
**Engaged in a conversation about FGM**		
No	-ref-	-ref-
Yes	1.4 (0.7–2.5)	0.7 (0.4–1.3)
**Social support (sought advice or instrumental support surrounding FGM)**		
No	-ref-	-ref-
Yes	0.6 (0.3–1.5)	0.7 (0.3–1.6)
**Descriptive norms**		
**Family decision to abandon FGM**		
No	-ref-	-ref-
Yes	0.1 (0.002–3.4)	0.3 (0.01–5.5)
**Other people in the community decide to abandon FGM**		
No	-ref-	-ref-
Yes	0.4 (0.01–11.0)	3.7 (0.1–96.3)
**Society in general decides to abandon FGM**		
No	-ref-	-ref-
Yes	4.4 (0.1–157.3)	*0.1 (0.01–1.3)^∧^*
**Injunctive norms**		
**Family expects you to abandon FGM**		
No	-ref-	-ref-
Yes	3.2 (0.2–52.4)	43.6 (2.7–687.8)^**^
**Other people in the community expect you to abandon FGM**		
No	-ref-	-ref-
Yes	20.6 (0.4–1,021.2)	0.2 (0.01–7.5)
**Society in general expect you to abandon FGM**		
No	-ref-	-ref-
Yes	0.2 (0.01–4.8)	3.5 (0.3–37.1)
**Punishments identified with abandoning FGM**		
Yes	-ref-	-ref-
No	1.0 (0.5–1.9)	0.8 (0.5–1.4)
**Rewards identified with abandoning FGM**		
No	-ref-	-ref-
Yes	0.8 (0.4–1.5)	1.5 (0.9–2.6)
**Region**		
Addis Ababa	-ref-	-ref-
Afar	0.09 (0.02–0.5)^**^	0.3 (0.03–2.0)
Southern Nations, Nationalities and People's Regions	0.1 (0.05–0.3)^***^	0.3 (0.1–0.6)^**^
**Respondent**		
Adolescent girl	-ref-	
Female caregiver	0.6 (0.2–2.0)	-ref-
Female influential	1.5 (0.5–4.9)	2.9 (1.01–5.2)^*^
Female social network	1.1 (0.4–2.7)	1.4 (0.7–2.6)
**Age**		
10–19 years	-ref-	-ref-
20–35 years	0.4 (0.2–1.2)	0.8 (0.3–2.4)
36 and over	0.2 (0.1–0.7)^*^	0.7 (0.2–2.4)
**Marital status**		
Married or engaged	-ref-	-ref-
Widowed, divorced or separated	1.0 (0.4–2.4)	0.5 (0.2–1.1)^∧^
Single, never married	2.8 (1.1–7.0)^*^	0.6 (0.2–1.7)
**Religion**		
Christian (Orthodox, Protestant, Catholic)	-ref-	-ref-
Muslim	0.3 (0.1–1.02)^∧^	3.1 (0.4–22.3)
**Educational status**		
No formal education	-ref-	-ref-
Primary education	1.6 (0.7–3.7)	1.3 (0.7–2.7)
Secondary education and higher	2.1 (0.9–4.9)^∧^	1.1 (0.5–2.6)
**Socio-economic status**		
Tertile 1 (most poor)	-ref-	-ref-
Tertile 2 (poor)	1.4 (0.6–3.1)	0.6 (0.3–1.2)
Tertile 3 (least poor)	1.4 (0.5–3.6)	1.0 (0.4–2.6)

∧ *p ≤ 0.1*,

* *p ≤ 0.05*,

** *p ≤ 0.01*,

*** *p ≤ 0.001*.

*■: Adolescent girls not asked this question*.

5The next column looked at the relationship between personal knowledge, attitudes, IPC, SS, and SN on knowing a family member who has not undergone FGM. Higher knowledge vs. lower knowledge was associated with a significantly lower odds of knowing a family member who had not undergone FGM [0.3 (95% CI: 0.1–0.5)]; if the family expected you to abandon FGM, you had a greater odds of knowing a family member who had not undergone FGM [43.6 (95% CI: 2.7–687.8)]; coming from Southern Nations, Nationalities and People's Region was associated with a lower odds of knowing a family member who had not undergone FGM [0.3 (95% CI: 0.1–0.6)]. Being a female influential vs. female caregiver was associated with a higher odds of knowing a family member who had not undergone FGM [2.9 (95% CI: 1.01–5.2)].

## Discussion

FGM is a practice with both short- and long-term negative impacts for girls and women. After several decades of interventions, there is adequate evidence that FGM abandonment requires change at both the individual and societal levels (26, 27, 37, 38). This paper uses data from Ethiopia to operationalize a validated macro-level monitoring and evaluation framework, summarized under the acronym ACT (32) to establish linkages between social norms change and FGM abandonment. Hence adding to the small but growing literature on norm shifting interventions to address harmful traditional practices (4, 39).

The results from the socio-demographic and socio-economic status questions raise some important points worth discussing. The socio-demographic and social-economic status of respondents did not vary across regions, significant differences in age, marital status, and educational attainment can be attributed to the fact that the study included girls and women over the age of 10. Respondents excluded from the analysis were more likely to be young, never married social network contacts in the SNNP region. The role of social networks in promoting behavior change is well-documented (40). Previous health communication research on specific health topics, for example, HIV risk, adolescent smoking, and obesity treatment have clearly highlighted the importance of using social network theories in SBC interventions to address harmful practices (41–43), While the importance of social network data and analysis is widely appreciated, some of the literature on social networks and norms is based on computational models (44). The findings from this study provide additional support to the value of including social network models as integral parts of interventions addressing social norms.

It is possible that in the SNNP traditional society, young unmarried girls, though mentioned as contacts did not respond to all the questions included in the ACT conceptual model. While this finding corresponds to the current literature on theory and practice of social norms in low-income countries (45), it also brings to the fore the inherent challenges of conducting quantitative research with hard to reach and hidden populations, highlighting the need for alternative sampling methods (46); intensive training for data collectors (47) and utilization of participatory research methods (48).

Seven multivariate analyses were run to examine the relationships between constructs in the conceptual model. There were some significant differences by respondent type around the key constructs in the model. The results pertaining to knowledge and attitudes were reversed, fewer adolescents knew (understood) what FGM was when compared to caregivers, however, a significantly higher proportion of adolescent girls had more positive attitudes with regard to the links between FGM as a harmful gender normative practice and also beliefs that FGM was not a part of female gender identity. Limited knowledge around FGM coupled with positive attitudes toward abandonment is somewhat of a paradox. However, similar results were found in a recent systematic scoping review among health care professionals. Health care workers had limited knowledge of FGM and its health implications but some of them openly disapproved of the practice (49). Which in turn impacts their ability to provide counseling and services to communities, with many of them performing FGM in secret for cultural and financial rewards (49). The lack of knowledge among adolescent girls is important to address since most girls are cut between the ages of 10 and 14, interventions specifically designed to improve knowledge about the practice specifically the short and long term impacts of the practice among girls is likely to help them truly understand what FGM and provoke critical thinking around benefits or sanctions related to being cut, instead of simply expressing disapproval of the practice in an effort to appear politically correct. These results also support findings presented by Valente et al. (50) questioning the linear ranking of knowledge, attitudes, and practices in promoting behavior change and instead promoting six different permutations examining the cause and effect relationships between knowledge, attitudes, and practices (50).

While communication scholars question this linear ranking of behavior change resulting from changes in knowledge and attitudes, there is considerable agreement across the field on the efficacy of strategic communication interventions to promote both behavior and social change (51–53). A comprehensive literature review of fifty mass media campaigns by Quattrin et al. (54), found that over two-thirds of the programs reported at least one statistically significant improvement in outcomes associated with knowledge, attitudes or practices. Additionally, communication literature has documented the positive impact of multi-channel, contextually specific social and behavior change interventions by location, for example, in developing countries (55); by topic, for example, nutritional status, STI prevention and control, HIV and global epidemics (56–59) and different audiences, for example women, adolescents and health care professionals (60, 61).

Although the attitudes toward the relationship of FGM and religion did not vary among respondent types, over eight out of 10 adolescent girls disagreed that the performance of FGM was a religious duty. These results might indicate a divide among younger and older women, with older women, who are significantly more likely to have themselves undergone FGM, reporting positive attitudes toward the practice. This finding is important given the fact that adolescent girls are likely to have less autonomy and decision-making power in a given household. Interventions designed to address FGM should focus on changing attitudes of married caregivers and older women to serve as change agents (62). This strategy has already been successfully piloted in West Africa, through the grandmother project promoting gender norm change (63).

Among socio-demographic variables, region emerged as a predictor for interpersonal communication. Respondents in the urban center of Addis Ababa are less likely to discuss FGM. This finding can be explained by the fact that Addis Ababa is a multicultural environment with loose social connections between neighbors. Also, the prevalence of FGM is comparatively low in Addis Ababa. Reported rates of interpersonal communication around FGM with people whose opinions mattered to the respondents were significantly higher in Afar and SNNP, both of which are largely close-knit rural communities with closer social ties and also a high prevalence of FGM, reported. One important takeaway from this finding is that it is not enough to focus on interpersonal communication as a whole but examine closely the frequency and content of the discussions and dialogues around FGM. Unfortunately, this research did not include questions about the content of the communication, so it is impossible to describe whether the reported communication was supportive or dismissive of FGM abandonment. On social support, the levels of social support reported by all respondents, especially in the rural regions of Afar and SNNP was very high. Highlighting perhaps the collectivist nature of rural Ethiopian society.

Between two-thirds and three-quarters of the respondents reported that FGM abandonment was related to descriptive and injunctive social norms. At the same time while several respondents mentioned rewards associated with abandonment a similarly large number felt that there was no punishment (social sanctions) associated with FGM continuation. Higher levels of knowledge around FGM were related to negative outcome expectations, i.e., those with higher levels of knowledge were significantly more likely to identify social sanctions associated with FGM abandonment.

There were significant relationships between norms and attitudes. Those who believed that FGM is associated with gender and power dynamics, part of their culture or identity and prescribed as part of religion were likely to report higher levels of perceived prevalence of FGM (descriptive norms/empirical expectations) and also feel that their family, community and society, in general, expected them to support and practice FGM (injunctive norms/normative expectations). Additionally, interpersonal communication emerged as a positive factor with those engaged in interpersonal communication more likely to lean toward norms associated with abandonment. This finding lends credence to the idea many social and behavior change communication scholars have discussed. They contend that harmful practices flourish in the absence of communication, creating “pluralistic ignorance” (64, 65).

The overall findings show that ACT constructs are associated with positive social norms. It also allows us to recommend that interventions focusing on information dissemination should likely craft messages around knowledge of social sanctions associated with FGM continuation. Creating an environment where individuals have positive attitudes and believe that their families, friends, neighbors, and community members support abandonment and discuss FGM abandonment with people whose opinion matters to them are likely to produce positive results. Public declarations to abandon FGM, which have been used as a key intervention in many countries, provide one opportunity to do this (66). Leveraging interpersonal communication within social networks is also important. This pilot study emphasizes the importance of interventions, that cut across individual, interpersonal, and community factors in the social-ecological model.

### Limitations

As with any other study, there are numerous limitations to contend with. The data used here is from a pilot study designed to validate tools to measure social norm change associated with communication interventions, it does not provide generalizable or representative information on FGM in Ethiopia. Although designed to serve a monitoring and evaluation function, this data did not include information on any specific intervention. The sample size of respondents was small because the analysis was limited to only those female respondents who had themselves undergone FGM or had a family member who had undergone FGM. Adolescent girls were not asked the question of if anyone in their family had undergone FGM, this further decreased the sample of adolescent girls. Additionally, since norms are socially driven, it might be useful to consider how boys, men, and religious leaders would respond to the norms-related questions. Another limitation as noted earlier was that interpersonal communication although included as a significant mediator, did not include probes on the content of the discussion and dialogue around FGM. With regard to the model, given the cross-sectional nature of the data, we are not able to examine the bidirectional relationship between social norms and behaviors or measure the process of change in any meaningful way.

## Conclusions

Many academics, researchers, and practitioners have remarked on the difficulty of measuring norms and linking norm change to exposure and involvement with communication interventions. Despite the limitations noted above, this paper has allowed us to operationalize a conceptual model on social norms measurement, specifically examining how social and behavior change communication can be used as a mechanism for shifting norms around a given harmful practice. Now that this model has been developed and validated, it is likely to provide a foundation to study the direct and indirect impacts of social norms programming on changing harmful practices, such as FGM. Plans are currently underway to incorporate the ACT framework into the FGM program in Sudan, which will allow us to use the pilot data to monitor and evaluate change.

## Data Availability Statement

The raw data supporting the conclusions of this article will be made available by the authors, without undue reservation.

## Ethics Statement

The studies involving human participants were reviewed and approved by Drexel University. Written informed consent to participate in this study was provided by the participants' legal guardian/next of kin.

## Author Contributions

SS and AR discussed the data available and determined the objectives of the manuscript. AR did the analyses and wrote up the methods and results. SS wrote the introduction and discussion section. Both authors contributed to the article and approved the submitted version.

## Funding

This work was funded by research grants from UNICEF, headquarters in New York under contract # 43214515, 43269511, and 43288753.

## Conflict of Interest

The authors declare that the research was conducted in the absence of any commercial or financial relationships that could be construed as a potential conflict of interest.

## Publisher's Note

All claims expressed in this article are solely those of the authors and do not necessarily represent those of their affiliated organizations, or those of the publisher, the editors and the reviewers. Any product that may be evaluated in this article, or claim that may be made by its manufacturer, is not guaranteed or endorsed by the publisher.
